# A screening tool to prioritize public health risk associated with accidental or deliberate release of chemicals into the atmosphere

**DOI:** 10.1186/1471-2458-13-253

**Published:** 2013-03-21

**Authors:** David H Blakey, Marc Lafontaine, Jocelyn Lavigne, Danny Sokolowski, Jean-Marc Philippe, Jean-Marc Sapori, Walter Biederbick, Regine Horre, Willi B Marzi, Hisayoshi Kondo, Yumiko Kuroki, Akira Namera, Tetsu Okumura, Miyako Yamamoto, Mikio Yashiki, Peter G Blain, David R Russell, Susan M Cibulsky, David A Jett

**Affiliations:** 1 Chemical Events Working Group of the Global Health Security Initiative, Berlin, Germany; 2 Environmental Health, Science and Research Bureau, Health Canada, Ottawa, Canada; 3 Chemical Emergency Preparedness and Response Unit, Health Canada, Ottawa, Canada; 4 Poison Control Center, Hospices Civils de Lyon, Centre Antipoison, Lyon, France; 5 Department of Emergency Response and Preparedness, Health General Directorate (Health Ministry), France; 6 Centre for Biological Security, Robert Koch-Institut, Berlin, Germany; 7 Research, Technology & Public Health Protection, Federal Office of Civil Protection and Disaster Assistance, Bonn, Germany; 8 Civil Protection, Federal Ministry of the Interior, Bonn, Germany; 9 MHLW DMAT Bureau, National Disaster Medical Center, Tokyo, Japan; 10 Tsukuba Office, Japan Poison Information Center, Tsukuba, Japan; 11 Forensic Medicine, Hiroshima University, Hiroshima, Japan; 12 Office of Assistant Chief Cabinet Secretary for National Security and Crisis Management, Cabinet Secretariat, Government of Japan, Tokyo, Japan; 13 Division of Safety Information on Drug, Food and Chemicals, National Institute of Health Sciences, Setagaya, Tokyo, Japan; 14 Medical Toxicology Centre, Newcastle University, Newcastle-upon-Tyne, United Kingdom; 15 WHO-Collaborating Centre for Chemical Incidents. The Health Protection Agency, Cardiff Metropolitan University, Western Avenue, CF5 2YB, Cardiff, Wales, UK; 16 Health and Human Services, Office of the Assistant Secretary for Preparedness and Response, Washington, DC, USA; 17 NINDS, National Institutes of Health, Bethesda, MD, USA

**Keywords:** Chemicals, Public health, Risk assessment, Atmospheric releases, Screening tool, Disaster management cycle

## Abstract

The Chemical Events Working Group of the Global Health Security Initiative has developed a flexible screening tool for chemicals that present a risk when accidentally or deliberately released into the atmosphere. The tool is generic, semi-quantitative, independent of site, situation and scenario, encompasses all chemical hazards (toxicity, flammability and reactivity), and can be easily and quickly implemented by non-subject matter experts using freely available, authoritative information. Public health practitioners and planners can use the screening tool to assist them in directing their activities in each of the five stages of the disaster management cycle.

## Background

The Global Health Security Initiative (GHSI) is an informal network of countries that came together shortly after the September 11, 2001 attacks, to ensure exchange and coordination of practices within the health sector in confronting new threats and risks to global health posed by terrorism. Delegations of the GHSI include Canada, France, Germany, Italy, Japan, Mexico, the United Kingdom, the United States and the European Commission. The World Health Organization (WHO) serves as an observer. The principal purpose of the GHSI is to strengthen global health preparedness and response to threats of biological, chemical and radio-nuclear terrorism and pandemic influenza. This document, written by the Chemical Events Working Group (CEWG) of the GHSI, recognizes that chemicals, despite conferring many benefits, may pose significant acute and chronic health risks in the event of an accidental or deliberate release. The public health impact of such an event is potentially catastrophic. Therefore, it is vital that emergency planning be developed at local, regional, national and international levels to effectively manage and mitigate chemical releases. Because of the millions of distinct chemicals, it is not realistic to plan and prepare for all chemicals. Risk must be prioritized so that the chemicals of greatest concern provide the basis for subsequent prevention, emergency planning and preparedness, detection and alert, response and recovery activities.

### The world of chemicals

The chemical industry is one of the world’s largest economic sectors, producing organic and inorganic chemicals, plastics, synthetic fibres, pharmaceuticals and medicines, synthetic rubber, soaps, paints and coatings, pesticides, fertilizers and other agricultural chemicals [1]. In 2010 worldwide chemical sales were valued at 2,353 billion euros. China was the largest chemical producer (€575.3 billion), followed by the United States (€395.2 billion), Japan (€152.7 billion) and Germany (€141.6 billion). In the European Union, the chemical industry directly accounted for 1.1 percent of total gross domestic production and employed 1,157,000 persons [2]. As of 1 May 2012, the American Chemical Society (ACS) Chemical Abstracts Service (CAS) had assigned Registry Numbers (RN) to 66,515,886 distinct organic and inorganic substances. The CAS Online Chemical Catalogues File (CHEMCATS) contained listings of more than 19,000,000 commercially available chemicals and their worldwide suppliers [3]. These commercially available chemicals are produced in quantities ranging from milligrams to millions of metric tons. High production volume (HPV) chemicals, as defined by the Organization of Economic Co-operation and Development (OECD), are those chemicals produced or imported into OECD countries in excess of 1,000 metric tons per year. In 2007, 4637 chemicals were classified as HPV chemicals [4]. Examples of HPV chemicals produced in excess of 5 million metric tons in 2010 are given in Table 1[1].

**Table 1:** **Examples of HPV chemicals **[1]

**Organic chemicals**	**Inorganic chemicals**	**Plastics**	**Fertilizers**
benzene	chlorine	polyethylene	ammonia
ethylene	sodium carbonate	polypropylene	ammonium nitrate
methanol	sodium hydroxide	polystyrene	phosphate rock
propylene	sulphuric acid	polyvinyl chloride	phosphoric acid

The World Health Organization (WHO) describes a chemical incident as the uncontrolled release of a chemical, resulting in (potential) harm to public health and the environment. Chemical incidents can arise from human activities and from natural sources (e.g., volcanic eruption, earthquake, forest fire) [5]. Chemical incidents, resulting from human activity, can be accidental or deliberate. Accidental releases can occur at any location in the production, use, storage, disposal or transportation cycle of the chemical. Examples of accidental chemical incidents that resulted in immediate significant deaths, injuries and property and/or environmental damage are listed in Table 2. These incidents, especially the 1976 release of dioxin at Seveso, greatly influenced national and international regulations, with respect to the amounts of chemicals that could be stored in a given location, land use provisions and transport regulations [5-9].

**Table 2:** Examples of chemical incidents resulting in regulatory actions

**Accident location**	**Date**	**Type of event**	**Consequences**	**Actions**	**Ref.**
Nypro UK Ltd, Flixborough, UK	1 Jun 1974	Explosion and fire – release of 30 tonnes of cyclohexane resulting in a vapour cloud explosion	28 killed; 89 injured, damage for several km	Influenced Seveso 1 content	[6,7,34]
				Led to the UK Health & Safety at Work Act & establishment of UK Health & Safety Executive	
Hoffmann LaRoche, Seveso, Italy	10 Jul 1976	Runaway thermal reaction – toxic and corrosive chemical cloud formed, containing phenols, sodium hydroxide, and ~2 kg of 2,3,7,8-tetrachlorodibenzo-p-dioxin (TCDD)	Over 5,700 residents evacuated; 220,000 people under medical surveillance; 447 cases of skin lesions or chloracne; >3000 animals dead; 80,000 animals slaughtered; affected a 18 sq km area; 20 billion lire paid in compensation	Led to Seveso 1 Directive	[5-7]
Union Carbide India Ltd, Bhopal, India	3 Dec 1984	Runaway reaction – 30–40 tonnes of methyl isocyanate released which drifted over a crowded working class neighbourhood; no warning for people within the area surrounding the plant	2,500-6,000 deaths; >200,000 injured; >50,000 survivors experiencing chronic ailments such as pulmonary fibrosis, bronchial asthma, chronic obstructive pulmonary disease, emphysema, recurrent chest infections, keratopathy and corneal opacities	Led to changes in Seveso I thresholds and proximity to residential populations, influenced land use planning provisions	[5-7,31,32]
				Led to USA Emergency Planning & Community Right to Know Act & CMA CAER Program	
Sandoz, Basel, Switzerland	1 Nov 1986	Warehouse fire – 30 tonnes of chemicals released into air and water (dinitro-ortho-cresol, organochlorines, organophosphates, ~150 kg mercury)	Massive contamination of the Rhine, 500,000 fish killed; pollution travelled over 500 km	Extended Seveso I to include storage activities	[6,7]
Phillips 66 Co, Pasadena, Texas, USA	23 Oct 1989	Explosion and fire – high density polyethylene production – release of >85,000 lbs of highly flammable process gases	23 deaths; more than 130 injured; over $1 billion in losses	Triggered 1990 USA Clean Air Act & Risk Management Program (RMP) & Process Safety Management (PSM) process standards	[6,34]
SE Fireworks, Enschede, The Netherlands	13 May 2000	Explosion and fire – 177 tonnes of fireworks exploded	22 killed; 947 injured; 2000 homes destroyed	Led to changes to definition of explosives in Seveso II	[5-7]
Aurul S.A., Baia Mara, Romania	30 Jan 2000	Breach in tailings dam – 100,000 m^3^ of cyanide rich tailings (cyanide plus heavy metals including copper) released into rivers feeding Danube and Black Sea	Contamination of water supply at 24 locations affecting 250,000 people; massive fish kill; destruction of aquatic species; pollution of ~ 200 km of river basin	Extended application of Seveso II	[6,7]
Grande Paroisse, Toulouse, France	21 Sep 2001	Explosion and fire – 300–400 tonnes of downgraded ammonium nitrate	30 deaths; 2,242 injured (20 seriously), 5,079 treated for stress; 25,000 homes damaged; 5 schools destroyed; 1,000 factories damaged; toxic chemicals leaked into river	Changed application of Seveso II with respect to ammonium nitrate	[5-7]

In addition to their legitimate use in industry, agriculture and medicine, chemicals have been used in warfare, by insurgents and terrorists. The direct use of chemicals, especially chlorine, phosgene and sulphur mustard, in World War 1 caused 91,198 deaths and 1,205,655 non-fatal injuries [10]. Since World War 1 additional chemical warfare agents, including the organophosphorus G series (e.g., sarin, soman, tabun) and V series (e.g., VX) of nerve agents have been developed. Sulphur mustard was used in the Iran-Iraq War of 1980–88, causing over 20,000 casualties [11]. The Chemical Weapons Convention (CWC), in effect since 1997, prohibits the use of chemical warfare agents, restricts the quantity that signatories may hold for research purposes and requires signatories to destroy existing stockpiles [12].

Deliberate chemical incidents occur when terrorists release a chemical in order to kill or injure humans or animals, to destroy crops or to cause extreme economic or environmental damage. Deliberate releases can occur at locations within the production, use, storage, disposal or transportation cycle of the chemical but also at totally unexpected locations. Terrorists have used reactive (explosive), flammable and toxic chemicals in their attacks. Transportation systems, especially subways and commuter rail lines, have been the principal targets as these afford easy access, have minimal security and are used by large numbers of people with luggage, bags and packages [13-15]. In 1994 Aum Shinrikyo became the first terrorist group to produce and use the nerve agent sarin when it released sarin outdoors in the city of Matsumoto, killing 7 individuals and injuring 262. In March 1995 Aum again released sarin, this time in the Tokyo subway, killing 12 individuals and causing 5,498 to seek medical attention [16].

Chemicals that consumers can purchase for home use, such as acids and alkalis, cleaners and pesticides are of concern. Hydrogen sulphide, which is produced by mixing readily available household chemicals [17], and phosphine, which is released by the action of water on phosphide fumigants and rodenticides (e.g., aluminium phosphide, zinc phosphide) [18,19], are widely used in suicides. The rodenticide, tetramethylene disulphotetramine (TETS), has been implicated in several homicides [20]. The inclusion of toxic chemicals as ingredients in food, beverages and consumer products continues to cause deaths and serious injuries (e.g., contaminated cooking oil [21], diethylene glycol in medications [5,22] and melamine in milk powder [23]).

### Scoping the problem

As shown above, chemicals are produced, used, stored, disposed of and transported widely and have the potential to harm the health of the public as a consequence of both acute and chronic health effects. Therefore, it is essential that countries develop emergency plans and prepare for chemical incidents at the local, regional, national and international level. Prioritization of risk is essential if resources are to be used efficiently. Hazards must be identified, risks prioritized and risk reduction strategies developed. Having a well-developed plan for risk prioritization and risk reduction can help adapt and focus preparedness efforts on chemicals of greatest concern for a given jurisdiction and ultimately, reduce casualties and hasten recovery [5].

### Development of a screening tool

The CEWG developed the following screening tool to prioritize the risk posed by the accidental or deliberate release of chemicals into the atmosphere. This tool is consistent with the following WHO statements that:

1. A release of a gas or aerosol into the atmosphere, resulting in an inhalational exposure, is likely to cause the maximum number of casualties [5]

2. Chemical incidents can cause injury through four basic injury mechanisms (fire, explosion, toxicity and the experience of traumatic events) [5]

The tool is semi-quantitative, independent of site, situation and scenario and encompasses all chemical hazards (toxicity, flammability and reactivity). CEWG considered it essential that the tool be easily and quickly implemented by non-subject matter experts using freely available, authoritative information. Chemical warfare agents and industrial chemicals (HPV, specialty, pharmaceuticals and pesticides) have been considered but toxins, even if they could be synthesized, have not.

#### Definition of risk

Risk is defined as the likelihood of harm occurring. CEWG used the definition of risk given in the Global Harmonized System of the Classification and Labelling of Chemicals [24]. This definition is general, not dependent on a particular scenario or situation and encompasses all chemical hazards.

(1) $$\mathrm{Risk}=\left(\mathrm{severity}\;\mathrm{of}\;\mathrm{hazard}\right)\times \left(\mathrm{probability}\;\mathrm{of}\;\mathrm{exposure}\right)$$

#### Determining severity of hazard

Hazard by definition refers to an inherent property of an object, place or situation that makes it potentially dangerous. In the context of chemicals, it is the degree of a chemical’s capacity to harm by interfering with normal biological processes and its capacity to burn, explode, corrode, produce toxicological effects, etc. Hazard is an intrinsic property of the chemical that cannot be modified. Chemical hazards are usually divided into three categories: toxicity, flammability and reactivity, all of which can be quantified [25]. Some chemicals can present more than one hazard, e.g. hydrogen sulphide is both toxic and flammable [24].

The severity of hazard is defined as the maximum hazard posed by the chemical.

(2) $$\mathrm{Severity}\;\mathrm{of}\;\mathrm{hazard}=\left(\mathrm{maximum}\;\mathrm{hazard}\;\mathrm{posed}\;\mathrm{by}\;\mathrm{the}\;\mathrm{chemical}\right)$$

For toxic chemicals, airborne releases can result in both inhalational and dermal exposures. Since inhalational exposures would most likely cause the maximum number of casualties [5], acute inhalation toxicity can be used as the toxicity parameter. When available, Acute Exposure Guidelines (AEGLs), developed by the United States Environmental Protection Agency (EPA), were used as the acute toxicity parameter. AEGLs represent threshold airborne exposure limits that are protective of public health and are applicable to emergency exposure periods ranging from 10 minutes to 8 hours. The AEGL-3, which is defined as the airborne concentration of a substance above which it is predicted that the general population, including susceptible individuals, could experience life-threatening health effects or death, was selected as the measure of the toxicity hazard [26]. When an AEGL-3 value was not available a Protective Action Criteria (PAC) value, developed by the United States Department of Energy, was used [27]. Several different toxicity scoring schemes [24,28,29] were considered before the one given in Table 3 was agreed upon. CEWG used the United States National Fire Protection Association (NFPA) criteria and scoring for flammability and reactivity hazards [30].

**Table 3:** Severity of hazard criteria and scoring of chemicals

**Inhalational toxicity**		** Flammability**		** Reactivity**	
**AEGL-3 or PAC-3 (mg/m**^**3**^**) for 60 min exposure**	**Toxicity score**	NFPA flammability criteria*	**NFPA score**	NFPA reactivity criteria*	**NFPA score**
≤1	4	Flammable gas or cryogenic material	4	Materials with instantaneous power density (IPD) of 1000 W/mL or greater @ 250°C; sensitive to localized thermal or mechanical shock at normal temperature and pressure	4
		Liquid with flash point (FP) below 22.8°C and boiling point (BP) below 37°C			
		Materials that spontaneously ignite when exposed to air			
>1, ≤10	3	Liquids with FP below 22.8°C and BP at or above 37.8°C; or FP at or above 22.8°C and below 37.8°C	3	Materials with IPD at or above 100 W/mL and below 1000 W/mL @ 250°C; sensitive to thermal or mechanical shock at elevated temperature and pressure	3
>10, ≤100	2	Liquids with FP at or above 37.8°C and below 93.4°C	2	Materials with IPD at or above 10 W/mL and below 100 W/mL @ 250°C	2
>100, ≤1000	1	Liquids, solids, semi-solids with FP above 93.4°C	1	Materials with IPD at or above 0.01 W/mL and below 10 W/mL @ 250°C	1
>1000	0	If assigned 0 by NFPA	0	Materials with IPD below 0.01 W/mL @ 250°C	0

* See NFPA 704 for complete listing of criteria [30].

The maximum hazard posed by a chemical is based on the highest score it received in one of the three hazard categories (inhalational toxicity, flammability and reactivity). The severity of hazard classes and scoring are given in Table 4.

**Table 4:** Severity of hazard classes and scoring

**Severity of hazard class**	**Extreme**	**Major**	**Significant**	**Moderate**	**Minor**
**Severity of hazard scoring**	4	3	2	1	0
(highest score received in one of the 3 hazard categories: flammability, toxicity, reactivity)					

This approach to determining severity of hazard is very informative as all hazards posed by a given chemical are clearly indicated. It is also flexible in that users can focus on a specific hazard category (e.g., inhalational toxicity) if they so desire.

#### Determining probability of exposure

The ease of release, either accidentally or deliberately, and the availability of the chemical can be used to estimate the probability of exposure [28,29].

(3) $$\mathrm{Probability}\;\mathrm{of}\;\mathrm{exposure}=\left(\mathrm{ease}\;\mathrm{of}\;\mathrm{release}\right)\times \left(\mathrm{availability}\right)$$

Airborne releases have the potential to cause massive casualties as once the chemical is released it has the potential to spread over a large area with little or no warning. Furthermore, unlike contaminated manufactured food or consumer products, airborne releases have zero possibility of recall. The Bhopal incident is an extreme example of casualties caused by a large airborne release of a toxic chemical [5,31,32]. The release of a highly flammable vapour cloud resulted in the explosions and fires in the Flixborough [6,33] and Pasadena Phillips 66 incidents [6,34]. Since the ease of creating an airborne release is directly related to the vapour pressure of the chemical, vapour pressure can be used as an indicator of ease of release. Criteria and scoring for determining the ease of airborne release of a chemical, which are similar to those used in ITF-25 [28], are given in Table 5.

**Table 5:** Vapour pressure scoring

**Vapour pressure (kPa @ 20°C)**	**Vapour pressure (mm Hg @ 20°C)**	**Score**
gas or pressurized liquid	gas or pressurized liquid	6
liquid, vp ≥ 50	liquid, vp ≥ 376	5
liquid, vp ≥ 10, <50	liquid, vp ≥ 75.2, <376	4
liquid/solid, vp ≥ 1, <10	liquid/solid, vp ≥ 7.52, <75.2	3
liquid/solid, vp ≥ 0.1, <1	liquid/solid, vp ≥ 0.752, <7.52	2
liquid/solid, vp <0.1	liquid/solid, vp <0.752	1

As a general rule, the greater the availability of the chemical, the more likely it will be involved in a chemical release event [35]. Consequently, chemicals that are widely produced, used, stored or transported are more likely to be involved in releases than those that have limited or specialized use. HPV chemicals are most readily available in large quantities. Many other commercially available chemicals have wide use but in much more limited quantities.

For many potential deliberate release scenarios using toxic chemicals, the quantity of chemical required to successfully execute the scenario is modest, ranging from grams to 100 kilograms, especially if the release is in a confined space. Terrorists most likely will choose to use readily available toxic, flammable or explosive chemicals or those that can be easily produced from readily available chemicals [36]. However, terrorists may choose to use synthesized or imported chemical warfare agents [16].

Criteria for determining the availability of chemicals are given in Table 6. The criteria are situationally independent as the general availability of the chemical rather than its availability in a specific location is considered. CEWG suggests that public health authorities undertake a detailed determination of all chemicals produced, used, stored, disposed of or transported through their area of responsibility so that the actual local/regional availability of the chemicals can be known. This survey would also note the location of each chemical, the quantity at that location, the state and security of the location, the adjacent population density and location of vulnerable facilities such as schools and hospitals.

**Table 6:** Criteria for determining the availability of chemicals and scoring

**Availability criteria**	**Availability score**
**H**igh **P**roduction **V**olume chemical, few purchase restrictions, widely used and transported, minimum security (**HPV**)	5
**C**ommercially **A**vailable, **N**o (or few) purchase restrictions, widely used, minimum security (**CAN**)	4
**C**ommercially **A**vailable, major purchase **R**estrictions, limited use, tight security (**CAR**)	3
not commercially available, **C**hemical **S**ynthesis easy, available precursors, standard equipment (**CS**)	2
not commercially available, **C**hemical **S**ynthesis **D**ifficult (complex multistep), special equipment (**CSD**)	1

The probability of exposure is determined according to equation 3. The probability of exposure classes and scoring are given in Table 7.

**Table 7:** Probability of exposure classes and scoring

**Probability of exposure class**	**Frequent**	**Likely**	**Occasional**	**Seldom**	**Unlikely**
**Probability of exposure scoring**	30-25	24-19	18-13	12-7	6-1

#### Determination of risk

**Figure 1:**
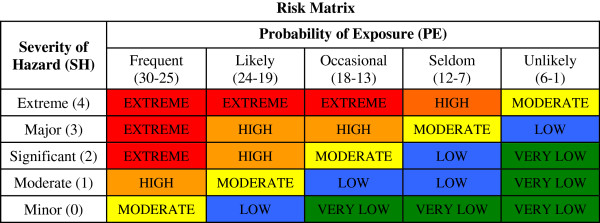
Risk matrix.

Several risk matrices were considered [29] before the five by five symmetrical matrix illustrated in Figure 1 was chosen. This matrix which gave the required degree of granularity was used to determine risk.

#### Validation of the screening tool

**Figure 2:**
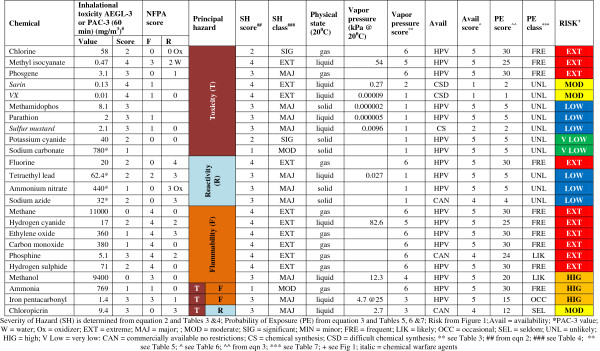
Example showing determination of risk for chemicals released into the atmosphere.

Chemicals used to test the tool were from Tables 1 and 2, the EU: List of Chemicals and Thresholds Seveso II Directive [7,8], the United States: List of Chemicals and Thresholds Risk Management Plan (RMP) Program (Sec. 68.130) [9] and the US Department of Homeland Security list [37]. The results of the testing are given in Figure 2.

As expected HPV gases and high vapor pressure liquids, that are highly toxic, flammable or reactive, are ranked extreme risk (e.g., chlorine, hydrogen cyanide, methane, fluorine). Highly toxic solids, that primarily constitute an ingestion hazard, are ranked low to very low risk (e.g., sodium azide, potassium cyanide). Chemical warfare agents, although extremely toxic, are ranked moderate or low because of their low vapor pressure and difficulty in synthesis (e.g., sarin). In addition to the chemicals shown in Figure 2, the tool has been used to rank several hundred chemicals of potential concern. The rankings are consistent with those observed in previous studies [28,29,38].

Detailed instructions on using the tool are given in Additional file 1: Guide to using CEWG tool to determine risk.

### The role of the public health community in the chemical disaster management cycle

The chemical risk prioritization tool presented in the previous sections allows for rapid screening of chemicals of greatest public health concern. However, ultimately, impacts and residual risk are situationally dependent. When planning for accidental releases, several measures such as conducting a survey of chemicals produced, used, stored, disposed of and transported through the area of concern combined with population data, allow public health practitioners to estimate the quantity of chemical that could be released and the number of individuals that could potentially be exposed and their duration of exposure. When planning for deliberate releases, additional measures such as ease of importing or producing an extremely hazardous chemical and identification of locations where release of the chemical could cause maximum harm must be considered. Ideally, all factors collectively designed to reduce the likelihood of a chemical release and to manage the release and impacts, should be considered to determine residual risks and assess vulnerabilities.

CEWG, in considering the role the public health community could play in preventing chemical incidents and minimizing the negative impacts of incidents on the exposed population and the environment, concluded that the public health community has a vital role within each of the five stages of the disaster management cycle (prevention, emergency planning and preparedness, detection and alert, response and recovery). The exact role will depend on the jurisdiction (local, regional, national, international) and the roles and capabilities of the other partners (industry, labour, government, international organizations) [5,6,39,40].

The first stage, prevention, focuses on reducing the likelihood of a chemical incident occurring and using all possible means (both organizational and technical) to reduce the severity of the incident if it does occur and to minimize its impact [5]. The public health community, as a critical component of an integrated emergency management structure, is essential in identifying hazardous chemicals, determining all possible release scenarios for these chemicals and assessing the health impact, both immediate and long term, from these scenarios. This includes determining the adequacy of data required for health impact assessments [26] and proposing research to fill critical data gaps. With respect to land use planning regulations governing the location of chemical production, use, storage and disposal sites and transportation infrastructure (ports, roads, rail lines, pipelines), the public health community can support legislation to ensure that these sites and corridors are located and built so as to minimize the risks to human health, the environment and property if a release occurs and can ensure that schools, hospitals and other major health facilities are located outside of potential hazard zones. The public health community can encourage industry to improve plant and equipment design and to replace hazardous chemicals and processes with less hazardous, but equally effective ones. Educating the public to demand and use less hazardous chemicals and ensuring that commonly used hazardous chemicals (e.g., pesticides and cleaners) and their containers are clearly and appropriately marked indicating health hazards so that they are not misused [5,41] are vital public health functions.

The second phase, emergency planning and preparedness, ensures that the negative outcomes of a chemical incident are minimized by responding to the emergency in a timely, appropriate and integrated way. The public health community can contribute to the design, set-up and maintenance of effective emergency response infrastructures with clearly defined roles and responsibilities for each participating group and to the development of chemical emergency plans covering detection, alert, command and control, training and exercises, public crisis communication and health sector communication. It has the major responsibility in developing public health incident response plans and ensuring that these are integrated with the overall chemical emergency plans. The public health community can also be influential in the development and maintenance of databases, essential for immediate response, including those for national hazardous sites, chemical information and health sector capabilities. At the local level, the public health community can be responsible for conducting community impact assessments for the hazardous sites located in the community or region, based on scenario studies of possible releases, as identified in the national hazardous sites database. Furthermore, the public health community is essential in assessing the adequacy of existing medical countermeasures for high risk chemicals, in recommending research and development of new countermeasures where required and in ensuring that existing countermeasures are available for immediate use. The preparation of information on chemical hazards and countermeasures that can be taken in the event of a release and the communication of this information to the public is a necessary public health function. The public health community can contribute to the establishment of and participate in routine training programs and exercises that are indispensible components of preparedness and response to chemical incidents.

The third phase, incident detection and alert, is an ongoing activity to determine that a chemical incident has occurred and ensure the rapid alert required for a timely and appropriate response. The public health community can support the installation of detection and alarming systems at hazardous sites and can take the lead in developing and implementing methods that can assist in the detection of less obvious chemical incidents. These include training in the recognition of chemical incidents for public health officials, medical professionals, first responders and members of the community; the provision of a well publicized phone number and/or Internet connection to report incidents to the appropriate authorities; the establishment and maintenance of a routine population health surveillance program and environmental monitoring system and the implementation of an alert channel to rapidly mobilize required personnel.

The first step in the fourth phase, response, is termination of the release followed by preventing the spread of contamination and reducing exposure. Although the public health community is not normally involved in the termination of atmospheric releases, it has an important role in reducing the spread of contamination including the rapid assessment of incident control options, assessing the need for decontamination of exposed persons, ensuring that contaminated persons do not leave the hazard zone prior to decontamination and advising on personal protection equipment and measures. The public health community also functions in assessing possible immediate and long term health effects so that appropriate responses can be determined. In the case of large airborne releases the public health assessment is a critical factor in deciding between the options of sheltering-in-place and evacuation. During the incident, the public health community acts to disseminate essential information and advice to responders, the public, and the media. This information must be consistent, tailored to the needs of the particular group and be simple, timely, accurate, relevant and credible (STARC). Conducting investigations that assess effects on health or on the environment during an incident so as to offer the best possible advice on treatment and protection throughout the incident, registering potentially affected individuals as soon as possible following a chemical release and conducting epidemiological investigations are other important public health functions.

The fifth and final stage, recovery, includes clean-up, health monitoring, evaluation and other activities that are aimed at restoring the community or site to an acceptable condition and contributing to prevention of a recurrence. The public health community has a vital role in organizing health care, including mental health care, to treat victims and support them in regaining control of their lives. Depending on the incident, care and support may be required for many years. Conducting risk and health outcome assessments, including exposure, environmental and human health assessments, implementing remediation and restoration actions, collecting and compiling epidemiological data and tabulating and disseminating lessons learned are other important functions the public health community can undertake in the recovery stage.

Table 8 summarizes the role public health can play in the disaster management cycle.

**Table 8:** The role the public health community can play in the chemical disaster management cycle

**Prevention**	**Emergency planning and preparedness**	**Detection and alert**	**Response**	**Recovery**
▪ Identifying chemical hazards	▪ Contributing to the design, set-up & maintenance of effective emergency response infrastructures	▪ Supporting installation of chemical detection & alarm systems	▪ Activating the public health aspects of the incident management system	▪ Organizing health care, including mental health care, to treat victims & to support them throughout the recovery cycle
▪ Conducting risk assessment	▪ Contributing to the development of integrated chemical emergency plans	▪ Establishing methods to detect & report covert chemical incidents	▪ Making rapid assessments of incident control options	▪ Undertaking risk & health outcome assessments
▪ Determining health impact of all potential release scenarios	▪ Developing public health chemical incident response plans	▪ Developing chemical incident recognition training	▪ Advising and alerting health care services	▪ Implementing remediation and restoration actions
▪ Communicating data on chemical hazards to the general public	▪ Supporting the development of relevant databases	▪ Developing diagnostic technologies for chemical exposures	▪ Ensuring coordination & integration of public health response	▪ Collecting and compiling epidemiological data
▪ Supporting land use planning regulations	▪ Preparing information on chemical hazards & countermeasures and communicating this information to the public	▪ Providing phone and Internet connections to report incidents	▪ Conducting a best outcome assessment for both immediate & long-term actions.	▪ Evaluating emergency response
▪ Supporting reduction in quantities of chemicals stored	▪ Maintaining an inventory of existing medical countermeasures	▪ Developing population health & environmental surveillance systems	▪ Disseminating information and advice to responders, the public & the media	▪ Tabulating and disseminating lessons learned
▪ Supporting product substitution	▪ Developing improved medical countermeasures	▪ Developing incident alert systems	▪ Registering all exposed individuals & collecting samples to estimate exposure	
▪ Supporting improved plant & equipment design	▪ Developing training programs		▪ Conducting epidemiological investigations	
▪ Supporting increased security at chemical transport and storage facilities	▪ Planning and participating in chemical incident exercises			
▪ Supporting law enforcement and intelligence				

## Conclusions

A flexible screening tool for chemicals that present a risk when released into the atmosphere has been developed. Risk, determined using this screening tool, is general, independent of site, situation and scenario, applicable to accidental and deliberate releases into the atmosphere and takes all chemical hazards (toxicity, flammability, reactivity) into consideration. The tool is semi-quantitative and can be easily and quickly implemented by non-subject matter experts using freely available authoritative information.

The role that the public health community can play in the chemical disaster management cycle is described.

## Abbreviations

ACS: American Chemical Society; AEGL: Acute exposure guideline level; BP: Boiling point; CAS: Chemical Abstracts Service; CEWG: Chemical Events Working Group; CHEMCATS: CAS online chemical catalogues file; CWC: Chemical Weapons Convention; EPA: United States Environmental Protection Agency; FP: Flash point; GHSI: Global Health Security Initiative; HPV: High production volume; IPD: Instantaneous power density; ITF: International Task Force; NFPA: United States National Fire Protection Association; OECD: Organization of Economic Co-operation and Development; PAC: Protective action criteria; PE: Probability of exposure; RN: CAS registry numbers; SH: Severity of hazard; TETS: Tetramethylene disulphotetramine; VP: Vapour pressure; WHO: World Health Organization.

## Competing interests

The authors declare that they have no competing interests.

## Authors’ contributions

All authors have contributed to the first draft and commented on the final draft. The paper was assembled by Health Canada. All authors read and approved the final manuscript.

## Authors’ information

The authors form part of the Chemical Events Working Group of the Global Health Security Action Group and collectively have expertise in the fields of laboratory medicine, general medicine, toxicology, emergency planning, environmental toxicology and science and policy making. This has provided the basis for the unique tool described in this manuscript.

## Pre-publication history

The pre-publication history for this paper can be accessed here:

http://www.biomedcentral.com/1471-2458/13/253/prepub

## Supplementary Material

Additional file 1 **“Guide to using the CEWG chemical risk screening tool 23_01_2013.pdf”.** The guide contains detailed instructions on the use of the CEWG tool to determine the risk posed by chemicals released into the atmosphere. Click here for file
