# Supplementary material for: A screening tool to prioritize public health risk associated with accidental or deliberate release of chemicals into the atmosphere
Source: BMC Public Health. 2013 Mar 21;13:253. doi: 10.1186/1471-2458-13-253 (PMC3751112; doi:10.1186/1471-2458-13-253)
Supplement: Additional file 1 — “Guide to using the CEWG chemical risk screening tool 23_01_2013.pdf”. The guide contains detailed instructions on the use of the CEWG tool to determine the risk posed by chemicals released into the atmosphere. [file 1471-2458-13-253-S1.pdf]

Guide to using CEWG chemical risk screening tool

The tool is used to determine the risk from chemicals that could be released, accidentally or deliberately, to present an inhalation hazard. The tool has been developed for generic risk assessment and considers all chemical hazards (flammability, toxicity, reactivity).

The example, given in Table 6, is generic, not site specific. It contains chemicals from the EU: List of Chemicals and Thresholds Seveso II Directive [1], the United States: List of Chemicals and Thresholds Risk Management Plan (RMP) Program (Sec. 68.130) [2] and the US Department of Homeland Security list [3].

The principal data sources required to use the tool are:

- International Chemical Safety Cards (ICSC) [4]
- Hazardous Substances Data Bank (HSDB) [5]
- Cameo Chemicals [6]
- Acute Exposure Guideline Levels (AEGLs) [7]
- Protective Action Criteria (PAC) [8]
- OECD 2007 List of High Production Volume (HPV) Chemicals [9]
- Chemical Weapons Convention Schedules [10]
- NIOSH Pocket Guide to Hazardous Chemicals (PGHC) [11]
- 2008 Emergency Response Guidebook [12]
- WISER [13]
- International Uniform Chemical Information Database (IUCLID) [14]
- NIOSH Emergency Response Safety and Health Database (ERSHD) [15]
- Royal Society of Chemistry Chemspider (Chemspider) [16]
- DrugBank [17]

All data sources are freely available on the Internet at the URLs given in the references.

A single chemical can have a variety of names; for example, methanol has several synonyms including methyl alcohol, carbinol and wood alcohol. However, every chemical has a unique universal identifier, the CAS Registry Number (CAS RN) [18]. Chemicals, which are widely produced and transported, also have a four digit UN Identification Number (UN ID) [12]. The CAS RN is used throughout the process to ensure that the chemical is consistently identified at each step.

The guide was written assuming that the user was simply given a list of chemicals, identified by a commonly used name, for assessment[1].

A schematic showing the steps is given in Figure 1.

[1] If the list was developed as the result of a survey of chemicals produced, used, stored, disposed of or transported through the area of responsibility, the user may have obtained the CAS RN or UN ID plus considerable data from the labeling/packaging of the chemical and from the Material Safety Data Sheet (MSDS) included with the chemical. The user may also have data on the quantity of chemicals and their location in his area of responsibility.

Figure 1: Schematic showing determination of risk

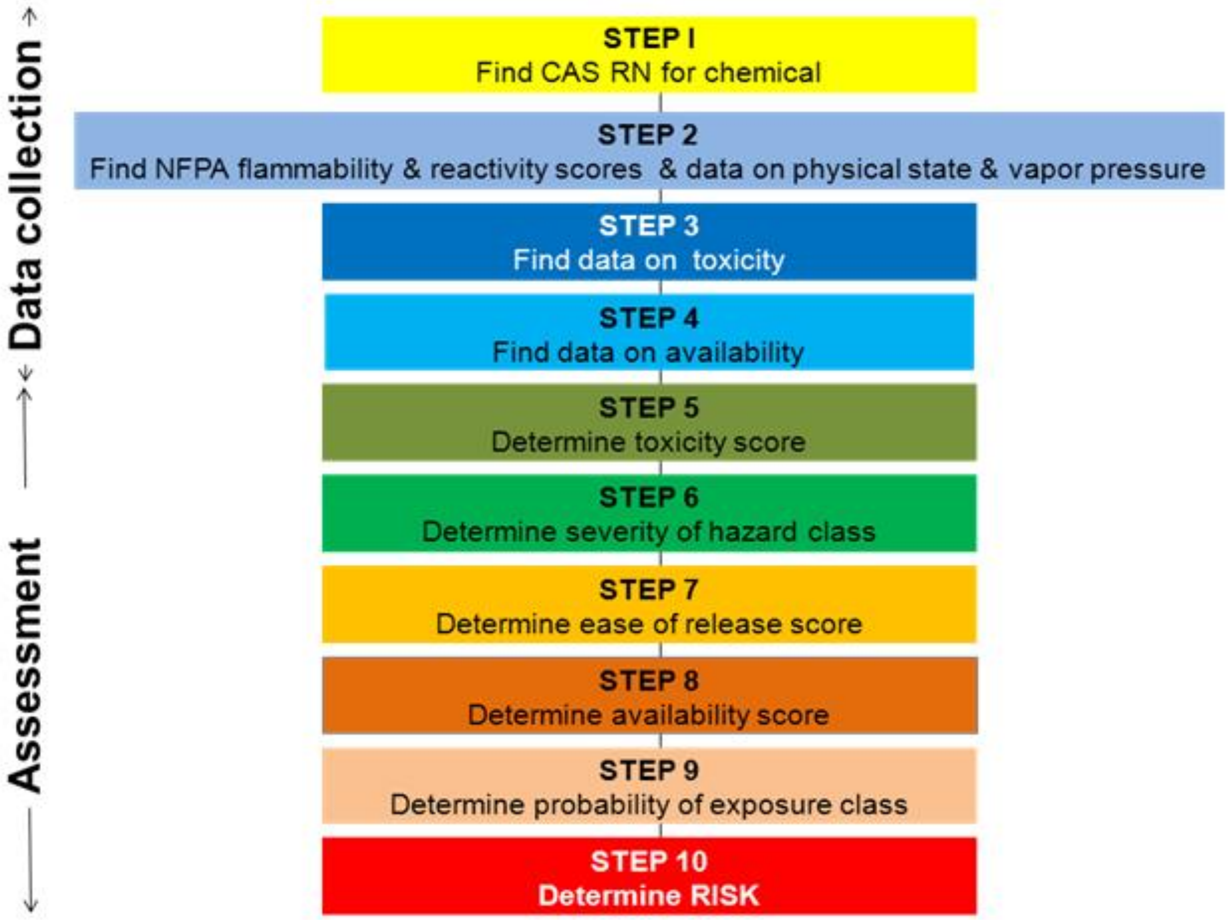

Step 1: Positively identify the chemical by obtaining the CAS RN. Start by using the ICSC [4], which can be searched by name, including synonyms, and several other identifiers[2]. If an ICSC is not available for the chemical, use the Protective Action Criterion Table 1: Chemicals of Concern and Associated Chemical Information PACs Rev 27, February 2012 (PAC Table1) [8], HSDB [5], Cameo [6], PGHC [11], ERSHD [15], ChemSpider [16] or by an Internet search[3] to obtain the CAS RN. Use the CAS RN, obtained in this step, to continue the assessment.

Step 2: Obtain data on the physical state, the vapour pressure and the NFPA rankings for flammability[4] and reactivity[5] from the ICSC [4], HSDB [5], Cameo [6], ERSHD [15], AEGL Technical Support Documentation [7] or other sources used in Step 1.

Step 3: Use the 60 minute AEGL-3 value as a measure of inhalational toxicity [7]. The value in mg/m^3 is given in the Technical Support Document provided for each chemical. If an AEGL

---

[2] There are several URLs for ICSCs: The INCHEM entry [http:www.inchem.org] provides access to the ICSCs plus other reports on chemicals in the International Program for Chemical Safety database. The NIOSH entry [http://www.cdc.gov/niosh/ipcs/icstart.html] allows searching by UN Number and has multilingual options.
[3] The Merck Index [19], which is **not** available free of charge on the Internet, can also be used.
[4] If the NFPA flammability score is not available, it can be calculated from data on boiling point and flash point according to the criteria given in Table 1, column 3. This should be done if the ICSC indicates that the chemical is flammable.
[5] If the NFPA reactivity score is not available and if the ICSC indicates that the chemical is highly reactive, a default value of 2 can be assigned.

value is not available, use the 60 minute PAC-3 given in Table 4: Protective Action Criteria (PAC) Rev 27 based on applicable 60-minute AEGLs, ERPGs, or TEELs [8] as the toxicity estimate[6].

Step 4: : Determine the availability of the chemical, first by determining if it is on the OECD 2007 List of High Production Volume (HPV) Chemicals [9][7]. If the chemical is not HPV, determine if it is commercially available by searching the Internet using the CAS Registry Number and asking for suppliers[8]. Drugs[9], some pesticides[10] and explosives[11], although commercially available, may be subject to purchase restrictions[12]. Chemical warfare agents (CWAs) are given in the Schedule 1 of the Chemical Weapons Convention [10]. CWAs are difficult to synthesis, requiring great technical expertise and good facilities, especially if kilogram or greater quantities are required[13].

---

6 If an AEGL-3 or PAC-3 is not available but other information indicates that the chemical is toxic, the chemical can be compared with similar chemicals that have an AEGL-3 or PAC-3 (e.g., pesticides can be compared with other pesticides). Additional sources of toxicity data include HSDB [12], ERSHD [15], INCHEM – International Program on Chemical Safety, Poisons Information Monographs [20], IUCLID [14], WISER [13]. Alternatively a default toxicity score of 2 can be assigned.

7 The OECD list of HPV chemicals includes those chemical produced or imported into the OECD countries in excess of 1,000 tonnes per year. Production of a given chemical by the large chemical industries of China, India and Brazil is only considered if those countries export that chemical in HPV quantities to an OECD country.

8 CAS Online Chemical Catalogs File (CHEMCATS) [18] contains data on over 19,000,000 commercially available chemicals and their worldwide suppliers. However, this catalogue is **not** free.

9 Data on over 6,000 drugs is given in DrugBank [17].

10 WHO Recommended Classification of Pesticides and Guidelines to Hazard 2009 provides data on widely used pesticides [http:www.int/ipcs/publications/pesticides_hazard/en/]

11 Lists of explosives are found in national export control documents (e.g., A Guide to Canada's Export Controls [http://www.international.gc.ca/controls-controles/assets/pdfs/documents/expoertcontrols2007-en.pdf] and in national regulations (e.g., Commerce in Explosives, List of Explosive Materials 2011R-18T, United States Department of Justice, [http://www.gpo.gov/fdsys/pkg/FR-2011-10-19/pdf/2011-26963.pdf])

12 Purchase restrictions are governed by national policy.

13 CWAs would be scored as 1. The exception is sulphur mustard (CAS RN 505-60-2) which would be scored as 2.

Step 5: Score toxicity according to Table 1.

Table 1: Severity of hazard criteria and scoring of chemicals

**Inhalational toxicity**		**Flammability**		**Reactivity**	
**AEGL-3 or PAC-3 (mg/m^3) for 60 min exposure**	**Toxicity score**	**NFPA flammability criteria***	**NFPA score**	**NFPA reactivity criteria***	**NFPA score**
<u><</u>1	4	Flammable gas or cryogenic material<br>Liquid with flash point (FP) below 22.8 ^oC and boiling point (BP) below 37 ^oC<br>Materials that spontaneously ignite when exposed to air	4	Materials with instantaneous power density (IPD) of 1000 W/mL or greater @ 250 ^oC; sensitive to localized thermal or mechanical shock at normal temperature and pressure	4
>1, <u><</u>10	3	Liquids with FP below 22.8 ^oC and BP at or above 37.8 ^oC; or FP at or above 22.8 ^oC and below 37.8 ^oC	3	Materials with IPD at or above 100 W/mL and below 1000 W/mL @ 250 ^oC; sensitive to thermal or mechanical shock at elevated temperature and pressure	3
>10, <u><</u>100	2	Liquids with FP at or above 37.8 ^oC and below 93.4 ^oC	2	Materials with IPD at or above 10 W/mL and below 100 W/mL @ 250 ^oC	2
>100, <u><</u>1000	1	Liquids, solids, semi-solids with FP above 93.4 ^oC	1	Materials with IPD at or above 0.01 W/mL and below 10 W/mL @ 250 ^oC	1
>1000	0	If assigned 0 by NFPA	0	Materials with IPD below 0.01 W/mL @ 250 ^oC	0

* see NFPA 704 for complete listing of criteria [21]

Step 6: Calculate the Severity of Hazard according to equation 2. The maximum hazard posed by a chemical is based on the highest score it received in any of the three hazard categories (inhalational toxicity, flammability and reactivity).

Severity of hazard = (maximum hazard posed by the chemical) (eqn 2)

Determine the severity of hazard class according to Table 2.

Table 2: Severity of Hazard Classes and Scoring

**Severity of Hazard Class**	Extreme	Major	Significant	Moderate	Minor
**Severity of Hazard Scoring**<br>(highest score received in any of the 3 hazard categories (flammability, toxicity, reactivity))	4	3	2	1	0

Step 7: Vapour pressure is used as an indicator of ease of release. Use the vapour pressure data[14] obtained in Step 2, to obtain the ease of release score according to Table 3

Table 3: Vapour pressure scoring

Vapour pressure (kPa @ 20 °C)	Vapour pressure (mm Hg @ 20 °C)	Score
gas or pressurized liquid	gas or pressurized liquid	6
liquid, vp≥50	liquid, vp≥376	5
liquid, vp≥10, <50	liquid, vp≥75.2, <376	4
liquid/solid, vp≥1, <10	liquid/solid, vp≥7.52, <75.2	3
liquid/solid, vp≥0.1, <1	liquid/solid, vp≥0.752, <7.52	2
liquid/solid, vp <0.1	liquid/solid, vp <0.752	1

Step 8: Score availability according to Table 4.

Table 4: Criteria for determining the availability of chemicals and scoring

Availability criteria	Availability score
**H**igh **P**roduction **V**olume chemical, few purchase restrictions, widely used & transported, minimum security (**HPV**)	5
**C**ommercially **A**vailable, **N**o (or) few purchase restrictions, wide use, minimum security (**CAN**)	4
**C**ommercially **A**vailable, major purchase **R**estrictions, limited use, tight security (**CAR**)	3
not commercially available, **C**hemical **S**ynthesis easy, available, precursors, standard equipment (**CS**)	2
not commercially available, **C**hemical **S**ynthesis **D**ifficult (complex multistep), special equipment (**CSD**)	1

Step 9: Determine the probability of exposure class using equation 3 and Table 5

Probability of exposure = (availability) X (ease of release) (eqn 3)

Table 5: Probability of Exposure Classes and Scoring

Probability of Exposure Class	Frequent	Likely	Occasional	Seldom	Unlikely
**Probability of Exposure Score**	30-25	24-19	18-13	12-7	6-1

Step 10: Determination of risk

Determine the risk from the risk matrix given in Figure 2.

[14] If vapour pressure data is not found in step 2, a default values of 1 can be assigned to solids and liquids, unless other information indicates that the liquid has a noticeable vapour pressure (e.g., odour) when 2 can be assigned.

Figure 2: Risk Matrix

Severity of Hazard	Probability of Exposure				
	Frequent (30-25)	Likely (24-19)	Occasional (18-13)	Seldom (12-7)	Unlikely (6-1)
Extreme (4)	EXTREME	EXTREME	EXTREME	HIGH	MODERATE
Major (3)	EXTREME	HIGH	HIGH	MODERATE	LOW
Significant (2)	EXTREME	HIGH	MODERATE	LOW	VERY LOW
Moderate (1)	HIGH	MODERATE	LOW	LOW	VERY LOW
Minor (0)	MODERATE	LOW	VERY LOW	VERY LOW	VERY LOW

An example is given in Table 6.

Table 6: Example showing determination of risk for chemicals released into the atmosphere

Chemical	CAS RN	Inhalational toxicity AEGL-3 (60 min) (mg/m^3)#		NFPA score		Principal hazard	Hazard score##	Hazard class###	Physical state (20^{0}C)	Vapor pressure (kPa @ 20^{0}C) **	Vapor pressure score^	Availability	Availability score ^^	Probability of exposure score^^^	Probability of exposure class^^^^	RISK+
		Value	Score	Flammability	Reactivity											
Chlorine	7782505	58	2	0	0 Ox	Toxicity (T)	2	SIG	gas		6	HPV	5	30	FRE	EXT
Methyl isocyanate	624839	0.47	4	3	2 W		4	EXT	liquid	54	5	HPV	5	25	FRE	EXT
Phosgene	75445	3.1	3	0	1		3	MAJ	gas		6	HPV	5	30	FRE	EXT
Aldicarb	116063	0.26	4				4	EXT	solid	0.00001 @25	1	CAN	4	4	UNL	MOD
Sarin	107448	0.13	4	1			4	EXT	liquid	0.27	2	CSD	1	2	UNL	MOD
VX	50782699	0.01	4	1	0		4	EXT	Liquid	0.00009	1	CSD	1	1	UNL	MOD
Methamidophos	10265926	8.1	3				3	MAJ	Solid	0.000002	1	HPV	5	5	UNL	LOW
Parathion	56382	2	3	1			3	MAJ	Liquid	0.000005	1	HPV	5	5	UNL	LOW
Sulfur mustard	505602	2.1	3	1	0		3	MAJ	Liquid	0.0096	1	CS	2	2	UNL	LOW
Potassium cyanide	151508	40	2	0	0		2	SIG	Solid		1	HPV	5	5	UNL	V LOW
Sodium carbonate	497198	780*	1				1	MOD	Solid		1	HPV	5	5	UNL	V LOW
Warfarin	81812	358*	1				1	MOD	Solid	0.133 @106	1	CAN	4	4	UNL	V LOW
Fluorine	7782414	20	2	0	4	Reactivity (R)	4	EXT	Gas		6	HPV	5	30	FRE	EXT
Tetraethyl lead	78002	62.4*	2	2	3		3	MAJ	Liquid	0.027	1	HPV	5	5	UNL	LOW
Ammonium nitrate	6484522	440*	1	0	3 Ox		3	MAJ	Solid		1	HPV	5	5	UNL	LOW
Sodium azide	26628228	32*	2	0	3		3	MAJ	Solid		1	CAN	4	4	UNL	LOW
Methane	74828	11000	0	4	0	Flammability (F)	4	EXT	Gas		6	HPV	5	30	FRE	EXT
Hydrogen cyanide	74908	17	2	4	2		4	EXT	Liquid	82.6	5	HPV	5	25	FRE	EXT
Ethylene oxide	75218	360	1	4	3		4	EXT	Gas		6	HPV	5	30	FRE	EXT
Carbon monoxide	630080	380	1	4	0		4	EXT	Gas		6	HPV	5	30	FRE	EXT
Phosphine	7803512	5.1	3	4	2		4	EXT	Gas		6	CAN	4	24	LIK	EXT
Hydrogen sulphide	7783064	71	2	4	0		4	EXT	Gas		6	HPV	5	30	FRE	EXT
Arsine	7784421	1.6	3	4	2		4	EXT	Gas		6	CAN	4	24	LIK	EXT
Methanol	67561	9400	0	3	0		3	MAJ	Liquid	12.3	4	HPV	5	20	LIK	HIG
Acrylonitrile	107131	217	1	3	2		3	MAJ	Liquid	11	4	HPV	5	20	LIK	HIG
Ammonia	7664417	769	1	1	0	T F	1	MOD	Gas		6	HPV	5	30	FRE	HIG
Iron pentacarbonyl	13463406	1.4	3	3	1	T F	3	MAJ	Liquid	4.7 @25	3	HPV	5	15	OCC	HIG
Chloropicrin	76062	9.4	3	0	3	T R	3	MAJ	Liquid	2.7	3	CAN	4	12	SEL	MOD
Carbon tetrachloride	56235	3270	0	0	0	T F R	0	MIN	Liquid	12.2	4	HPV	5	20	LIK	LOW
Chloroform	67663	16000	0	0	0	T F R	0	MIN	Liquid	21.2	4	HPV	5	20	LIK	LOW

Severity of Hazard is determined from equation 2 and Tables 3 &4; Probability of Exposure from equation 3 and Tables 5, 6 &7; Risk from Figure 1; *PAC-3 value; ** @20^{0}C unless indicated otherwise; W = water; Ox = oxidizer; EXT = extreme; MAJ = major; ; MOD = moderate; SIG = significant; MIN = minor; FRE = frequent; LIK = likely; OCC = occasional; SEL = seldom; UNL = unlikely; HIG = high; V Low = very low: CAN = commercially available no restrictions; CS = chemical synthesis; CSD = difficult chemical synthesis; # see Table 3; ## from eqn 2; ### see Table 4; ^ see Table 5; ^^ see Table 6; ^^^ from eqn 3; ^^^^ see Table 7; + see Fig 1; italic = chemical warfare agents

References

1. European Commission Environment: *Chemical Accidents (Seveso II) - Prevention, Preparedness and Response.* [http://ec.europa.eu/environment/seveso/index.htm]
   Council Directive 96/82/EC of 9 December 1996 on the control of major-accident hazards involving dangerous substances

2. United States Environmental Protection Agency: *Accidental Release Prevention Requirements: Risk Management Programs Under the Clean Air Act, Section 112(r)(7); List of Regulated Substances and Thresholds for Accidental Release Prevention, Stay of Effectiveness; and Accidental Release Prevention Requirements: Risk Management Programs Under Section 112(r)(7) of the* Clean Air Act as Amended, Guidelines; Final Rules and Notice. 61 FR 31667 (June 20, 1996): [http://www.epa.gov/fedrgstr/EPA-AIR/1996/June/Day-20/pr-23439.pdf]

3. Cox JA, Roszell LE, Whitmire M, **Chemical Terrorism Risk Assessment: A Biennial Assessment of Risk to the Nation**, United States Department of Homeland Security, Chemical Security Analysis Center, May 2010.

4. International Chemical Safety Cards (ICSC) INCHEM entry [http://www.inchem.org/]; NIOSH entry [http://www.cdc.gov/niosh/ipcs/icstart.html]

5. United States National Library of Medicine, *Hazardous Substances Data Bank (HSDB)* [http://toxnet.nlm.nih.gov/cgi-bin/sis/htmlgen?HSDB]

6. United States Department of Commerce, National Oceanic and Atmospheric Administration: *Cameo Chemicals-Database of Hazardous Materials* [http://cameochemicals.noaa.gov]

7. United States Environmental Protection Agency: *Acute Exposure Guidelines* [http://epa.gov/opptintr/aegl]

8. United States Department of Energy, Office of Health, Safety and Security: *Protective Action Criteria (PAC) with AEGLs, ERPGs, & TEELs: Rev. 27 for Chemicals of Concern (02/2012)* [http://www.hss.energy.gov/healthsafety/wshp/chem._safety/teel.html]
   Table 1: Chemicals of Concern and Associated Chemical Information PACs Rev 27, February 2012[http://www.atlintl.com?DOE/teels/teel/Table1.pdf]
   Table 4: Protective Action Criteria (PACs) Rev 27 based on applicable 60 min AEGLs, ERPGs or TEELS [http://www.atlintl.com/DOE/teels/teel/Table4.pdf]

9. Organisation for Economic Co-operation and Development: Environment Directorate, *Series on testing and assessment, Number 112: The 2007 OECD list of high production volume chemicals.* 2009. Paris. [http://www.oecd.org/officialdocuments/displaydocumentpdf/?cote=env/jm/mono(2009)40&doclanguage=en]

10. Convention on the Prohibition of the Development, Production, Stockpiling and Use of Chemical Weapons and on their Destruction (Chemical Weapons Convention) [http://www.opcw.org/chemical-weapons-convention]

11. Department of Health and Human Services, Centers for Disease Control and Prevention, National Institute for Occupational Safety and Health, *NIOSH Pocket Guide to Chemical Hazards* September 2007 [http://www.cdc.gov/niosh/doc)]

12. Transport Canada (TC), the U.S. Department of Transportation (DOT), the Secretariat of Transport and Communications of Mexico (SCT) *2008 Emergency Response Guidebook* [http://www.tc.gc.ca/eng/canutec/guide-ergo-221.htm]

13. WISER (Wireless Information System for Emergency Responders) [http://wiser.mlm.nih.gov]

14. International Uniform Chemical Information Database (IUCLID) [http://iuclid.eu.index.php?fuseaction=home.project]

15. United States National Institute for Occupational Health and Safety (NIOSH)*: The Emergency Response Safety and Health Database* [http://www.cdc.gov/NIOSH/ershdb]

16. Royal Society of Chemistry (RSC) ChemSpider [http//www.chemspider.com]

17. DrugBank [http://www.drugbank.ca]

18. Chemical Abstracts Service [http://cas.org]

19. *Merck Index: Encyclopedia of Chemicals, Drugs and Biological Series Fourteenth Edition,* (ISBN-13:9780911910001) John Wiley& Sons, Inc., 2006

20. INCHEM – International Program on Chemical Safety, Poisons Information Monograph [http://www.inchem.org/]

21. Colonna GR (Ed): *Fire Protection Guide to Hazardous Materials, 2010 Edition*. Quincy, Massachusetts: National Fire Protection Association; 2010.

All URLs were assessed on 18 July 2012.
